# Editorial: Challenges of Pharmacoeconomics in Global Health Arena

**DOI:** 10.3389/fpubh.2018.00368

**Published:** 2018-12-21

**Authors:** Mihajlo Jakovljevic, Nick Verhaeghe, Kyriakos Souliotis

**Affiliations:** ^1^Global Health Economics and Policy, Faculty of Medical Sciences, University of Kragujevac, Kragujevac, Serbia; ^2^Division of Health Economics, Lund University, Lund, Sweden; ^3^Department of Public Health and Primary Care, Interuniversity Centre for Health Economics Research, Ghent University, Ghent, Belgium; ^4^Faculty of Social and Political Sciences, University of Peloponnese, Corinth, Greece

**Keywords:** pharmacoeconomics, reimbursement, medicines, global, costs, policy, pharmaceutical, expenditure

The pace of globalization has significantly accelerated since the end of the Cold War Era in 1989. These changes profoundly affected health care systems worldwide (Jakovljevic et al., Jakovljevic et al.). Health policy makers increasingly started facing new harsh challenges in their uneasy task to provide universal health coverage and decent equity of access to medical services. Among the most prominent demand-side issues are extended longevity joined with population aging (1), rise of non-communicable diseases, and growing patient expectations (2). Supply-side causes are gains in societal welfare and living standards, technological innovation in medicine and continuing rapid urbanization in developing world regions (3). Successful insurance-based risk sharing agreements made drug dispensing and medical service provision cheap or virtually free at the point of consumption in most OECD and many middle-income countries. Coupled with massive build-up of workforce capacities and strengthening of primary care and hospital networks, all these factors contributed to the “supplier induced demand” phenomenon (4).

There is straightforward historical evidence of long-term growth in pharmaceutical and overall health spending both in absolute and GDP% terms worldwide (5). The accumulated constraints deriving from skyrocketing costs of care were felt in many areas of clinical medicine even among the richest societies (6). Cardinal examples of expensive and hardly affordable therapeutic areas are orphan drugs indicated to treat rare diseases and targeted biologicals used in autoimmune disorders and cancer (Kamusheva et al.). Last but not least, is troubled and frequently denied access to even essential generic pharmaceuticals still taking place in many nations (7). This appears to be particularly the case among the world's poor and underserved citizens residing in rural and suburban areas of low- and middle-income countries (3). To a large extent, these difficulties are worsened by lack of evidence-based resource allocation strategies and less sustainable financing strategies (Pejcic).

This Research Topic has successfully attracted a variety of contributions tackling the core challenges of medicines provision and medical care financing across the globe. Its target to reveal some of the hidden underlying causes of uneven access to medicines was achieved to great extent. A total of eleven articles have been published. Exceptional regional diversity covering national health system issues ranging from Papua New Guinea to Brazil, Syria, Denmark, Finland, Bulgaria, Serbia, Bosnia and Herzegovina, Croatia, Macedonia, Montenegro, Slovenia, and South Africa.

A variety of methodological approaches was exploited in these articles inclusive of epidemiological research, perspectives, literature reviews, commentaries, and ultimately two systematic reviews. Large part of these contributions focused on sustainability of antibiotics supply in hospitals (Zwane et al.) and clinical and economics consequences of irresponsible prescribing and dispensing (Horvat et al.). Probably the most prominent example, given the civil war related circumstances is the contribution coming from Syria, describing how one of the most developed pharmaceutical industries in MENA/Eastern Mediterranean region came to drug shortages of essential medicines (Jakovljevic et al.).

Japanese research was conducted on malaria diagnostics in pediatric South-East Asian populations (Tsukahara et al.). The Bulgarian group wrote an excellent review on the role of ethical and legal considerations in biometric data usage (Deliversky and Deliverska). Another piece coming from Balkan academic centers dealt with prescribing policies on pharmaceuticals and their affordability among chronic patients suffering from NCDs (Pekez-Pavlisko et al.). Probably the two most ambitious pieces were the two systematic reviews. The first one compiled the evidence published in the Brazilian academic, industry and governmental sectors output in interdisciplinary studies surrounding health economics (Decimoni et al.) while the second one, compiled by one of the Topic editors, did a bibliographic synthesis of global health economics publishing output in quantitative terms (Jakovljevic and Pejcic). Given the entire scale of contributions by solicited and unsolicited research groups worldwide, Editors believe that the Research Topic has lived up to its goal and achieved expectations filling some knowledge gaps in the science of pharmacoeconomics.

## Author Contributions

MJ, NV, and KS have jointly designed the research question, prepared the manuscript, and revised it for important intellectual content.

### Conflict of Interest Statement

The authors declare that the research was conducted in the absence of any commercial or financial relationships that could be construed as a potential conflict of interest.
